# Improvement of Sepsis Prognosis by Ulinastatin: A Systematic Review and Meta-Analysis of Randomized Controlled Trials

**DOI:** 10.3389/fphar.2019.01370

**Published:** 2019-11-26

**Authors:** Huifang Wang, Bin Liu, Ying Tang, Ping Chang, Lishuai Yao, Bo Huang, Robert F. Lodato, Zhanguo Liu

**Affiliations:** ^1^Department of Intensive Care Unit, Zhujiang Hospital, Southern Medical University, Guangzhou, China; ^2^Emergency Department, Zhujiang Hospital, Southern Medical University, Guangzhou, China; ^3^Department of Thoracic and Cardiovascular Surgical, Zhujiang Hospital, Southern Medical University, Guangzhou, China; ^4^Department of Pulmonary, Critical Care, and Sleep Medicine, Medical School, University of Texas Health Science Center at Houston, TX, United States

**Keywords:** sepsis, ulinastatin, mortality, inflammatory cytokine, immune system

## Abstract

**Background:**

Ulinastatin has been prescribed to treat sepsis. However, there is doubt regarding the extent of any improvement in outcomes to guide future decision making.

**Objectives:**

To evaluate the effects of ulinastatin on mortality and related outcomes in sepsis patients.

**Methods:**

Thirteen randomized controlled trials and two prospective studies published before September 1, 2018, that included 1358 patients with sepsis, severe sepsis, or septic shock were evaluated. The electronic databases searched in this study were PubMed, Medline, Embase, and China National Knowledge Infrastructure (CNKI) for Chinese Technical Periodicals.

**Results:**

Ulinastatin significantly decreased the all-cause mortality {odds ratio (OR) = 0.48, 95% confidence interval (CI) [0.35, 0.66], p < 0.00001, I^2^ = 13%}, Acute Physiology, Age, Chronic Health Evaluation II (APACHE II) score {mean difference (MD) = -3.18, 95%CI [-4.01, -2.35], p < 0.00001, I^2^ = 33%, and reduced the incidence of multiple organ dysfunction syndrome (MODS) (OR = 0.3, 95% CI [0.18, 0.49], p < 0.00001, I^2^ = 0%). Ulinastatin also decreased the serum levels of IL-6 (MD = -53.00, 95% CI [-95.56, -10.05], p = 0.02), TNF-a MD = -53.05, 95%CI [-68.36, -37.73], p < 0.00001, and increased the serum levels of IL-10 (MD = 37.73, 95% CI [16.92, 58.54], p = 0.0004). Ulinastatin administration did not lead to any difference in the occurrence of adverse events.

**Conclusions:**

Ulinastatin improved all-cause mortality and other related outcomes in patients with sepsis or septic shock. The results of this meta-analysis suggest that ulinastatin may be an effective treatment for sepsis and septic shock.

## Introduction

Sepsis is life-threatening organ dysfunction caused by a dysregulated host response to infection (Singer et al., 2016). It is the major cause of death in intensive care units (Martin et al., 2003). Epidemiological studies in the United States have shown that 750,000 cases are diagnosed with severe sepsis annually, and 215,000 deaths occur every year (Angus et al., 2001). Owing to advances in the management of sepsis, such as early fluid resuscitation, early administration of antibiotics, and advances in supportive care, such lung-protective mechanical ventilation, the risk of sepsis-associated death has been decreasing. However, the mortality of sepsis remains high (Stoller et al., 2016).

The mechanism of sepsis is complicated (Armstrong et al., 2017; Minasyan, 2017). Sepsis initiates a complex interplay of host pro-inflammatory and anti-inflammatory processes (Hotchkiss et al., 2013; Chousterman et al., 2017). Simultaneously, both inflammatory response and immunosuppression are involved in sepsis (Hotchkiss et al., 2013; Hotchkiss and Crouser, 2015). Serum concentrations of tumor necrosis factor-α (TNF-α), interleukin-6 (IL-6), interleukin-8 (IL-8), and many other cytokines or chemokines are increased after the onset of sepsis (Chousterman et al., 2017; Rajaee et al., 2018). Immunoparalysis caused by the apoptosis of many immune cells, including T cells, B cells, dendritic cells, and neutrophils, is another dominant problem in sepsis patients; this state in turn results in a depressed immune system and failure in the elimination of pathogens and maintenance of immune balance (Hotchkiss et al., 2013; Girardot et al., 2017). Furthermore, anti-apoptosis therapy by blocking receptors or inhibitors of apoptotic pathway can reduce mortality in sepsis models (Zhang et al., 2010; Harjai et al., 2013). Blocking of programmed cell death receptor-1 (PD-1) also demonstrated a potential toward the reduction of sepsis-associated mortality (Zhang et al., 2010; Hotchkiss et al., 2013; Patera et al., 2016).

It is known that serine proteases are involved in systemic inflammation and cell apoptosis (Wong, 1998; Wiedow and Meyer-Hoffert, 2005). Urinary trypsin inhibitor (also called ulinastatin or UTI) is an important protease inhibitor found in human urine, blood, and other tissues (Linder and Russell, 2014). It has been shown that UTI plays an anti-inflammatory role by decreasing the phosphorylation of p38 mitogen-activated protein kinase (p38-MAPK) and nuclear factor-κB (NF-κB) activation as well as an anti-apoptotic role by protecting the mitochondria and scavenging oxygen free radicals (Shu et al., 2014; Li et al., 2016). The study of UTI mechanism revealed that UTI can decrease the level of inflammatory mediators and reduce the frequency of immune cell apoptosis in sepsis models. Therefore, UTI has been proposed as a potentially new therapeutic option for the treatment of sepsis and multiple organ dysfunction syndrome (MODS) (Linder and Russell, 2014; Atal and Atal, 2016).

Recently, clinical trials in sepsis patients treated with UTI or UTI combined with thymosin α1 showed a survival benefit trend (Wu et al., 2013a; Karnad et al., 2014). Meanwhile, five meta-analysis of studies in sepsis patients either UTI administration alone or a combination of UTI and thymosin α1 have been published (Han et al., 2015; Li et al., 2015; Feng et al., 2016; Wang et al., 2016; Liu et al., 2017). Among these five meta-analysis, two meta-analysis (Feng et al., 2016; Liu et al., 2017) analyzed the effect of using UTI alone and no significant difference between UTI group and control group in the 28-day mortality. Given that the results of these two meta-analysis are based on subgroup analysis, which included the same two trials (Wu et al., 2013a; Karnad et al., 2014), it is difficult to prove the effect of UTI alone. At present, it remains unclear whether the beneficial impact is rendered by UTI, thymosin α1, or the combination. Therefore, we pooled the randomized controlled trials that involved the use of UTI alone in order to clarify the efficacy of UTI in sepsis.

## Materials and Methods

**Figure 1:**
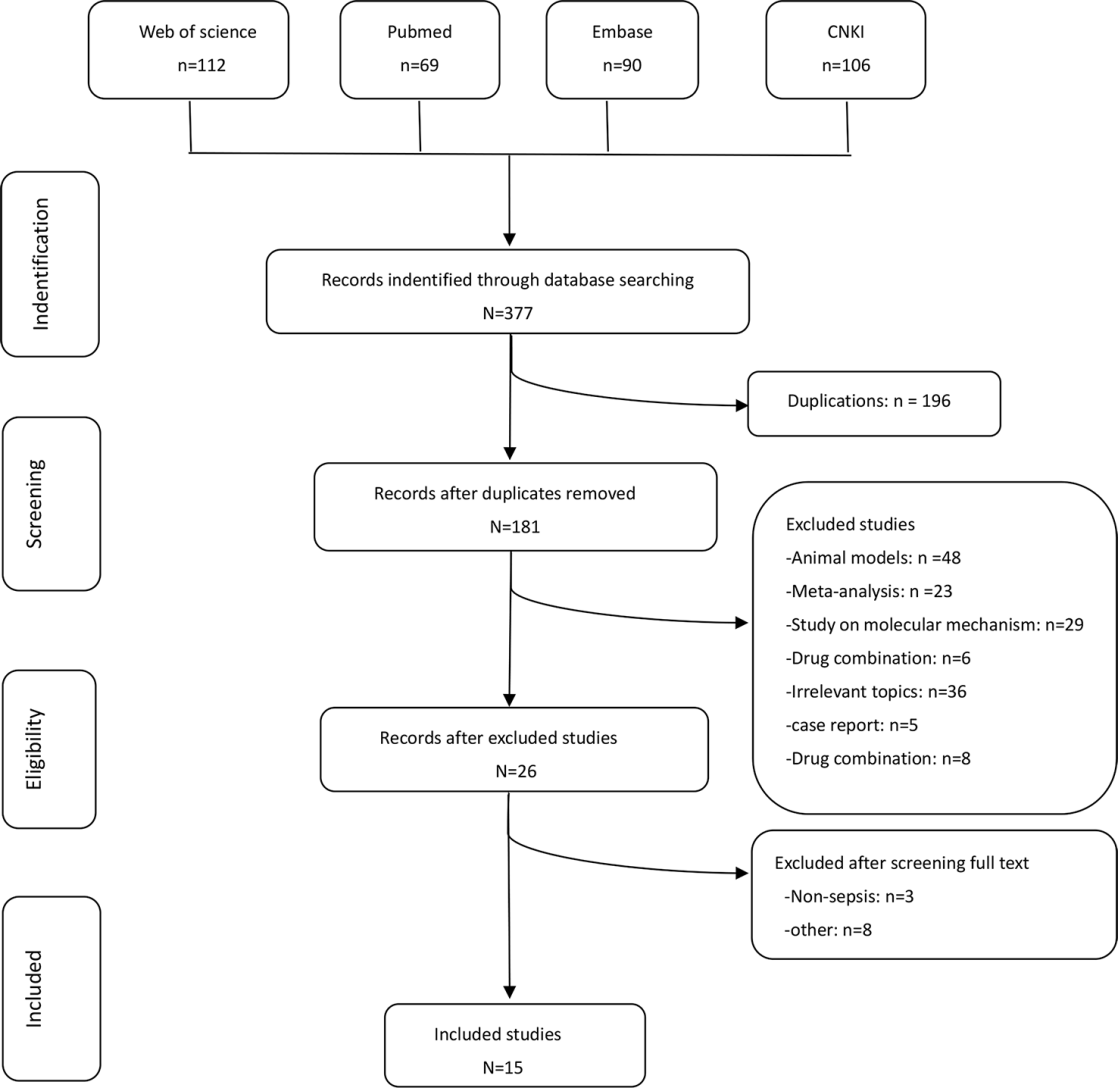
Flow diagram of the study selection process.

In accordance to the PRISMA (Preferred Reporting Items for Systematic Reviews and Meta-Analysis) statement for reporting systematic reviews and meta-analysis (Liberati et al., 2009), two groups of the present authors [(HW and LY) and (YT and BH)] independently conducted literature searches, established the study inclusion and exclusion criteria, performed quality assessment, and extracted data. If a consensus was not reached, it was resolved by the senior authors (ZL and PC). The flow graph is shown in **Figure 1**.

### Study Registration

Registration number in PROSPERO: CRD42018110751, an international prospective register of systematic reviews.

### Search Strategy

We searched for randomized controlled trials in sepsis published on or before September 01, 2018, regardless of language, publication type, or study region. The electronic database includes PubMed, Medline, Embase, and China National Knowledge Infrastructure (CNKI). The key words that were searched and their combinations in (title/abstract) are shown in **Table 1**.

**Table 1:** Search terms and phrases used in the meta-analysis.

#1 ulinastatin
#2 UTI
#3 urinary protease inhibitor
#4 sepsis
#5 sept*mia
#6 effect*
#7 treatment
#8 therap*
#9 Systemic Inflammatory Response Syndrome
#10 SIRS
#11 MODS
#12 Multiple organ dysfunction syndrome
#11 #1or #2 or #3
#12 #4 or #5 or #9 or #10 or #11 or #12
#13 #11 and #12 and #6
#14 #11 and #12 and #7

The means of "*" is truncation.

### Selection Criteria

Studies were included if they met the following criteria: 1) participants: patients who were diagnosed with sepsis, severe sepsis, or septic shock; 2) type of interventions: use of UTI alone regardless of treatment duration; 3) research design: either randomized controlled trial (RCT) or prospective cohort study. Review articles, animal experimental studies, case reports, and letters that did not describe outcomes or were not published as full reports were excluded. In addition, we only included the most updated and completed studies in case of duplicated publication.

### Outcomes and Data Extraction

The primary outcome was all-cause mortality. The secondary outcomes were changes in the serum levels of IL-6, IL-10, and TNF-α, the incidence rate of MODS, and changes in Acute Physiology, Age, Chronic Health Evaluation II (APACHE II) scores. We also collected the following information: study design, year of study, country, study period, the number of patients included, intervention methods, and adverse events. The main characteristics of the included studies are shown in **Table 2**. For the continuous variables, we acquired data according to the following method. For calculating the mean in this meta-analysis, we employed the formula X = X_2_ - X_1_, where X represents the mean applied in this meta-analysis, X1 represents the baseline mean, and X2 represents the endpoint mean. For calculating the standard deviation (SD) in this metaanalysis, we chose to employ the formula $S^{2}=S_{1}^{2}+S_{2}^{2}-2\times R\;\times S_{1}\;\times S_{2}$, where S represents the standard deviation applied in this meta-analysis, S1 represents the baseline SD, and S_2_ represents the endpoint SD. R = 0.5 in the meta-analysis, which was described in the Cochrane Handbook. All data were independently extracted by two authors (HW and LY). HW entered data into the computer and LY checked them.

**Table 2:** The characteristics of the included studies.

Author (year published)	Country	Study period	Study Type	Total No. of patients	Number of patients		Mean Age, yrs		Diagnosis	Interventions		Outcomes		Adverse effects	Quality score
					UTI group	Control group	UTI group	Control group		UTI group	Control group	Primary outcome	Secondary outcomes		
Fang and Zhao, (2017)	China	2013.03-2015.05	RCT	96	49	47	56.7 ± 12.5	59.3 ± 11.6	severe sepsis	30,0000 IU q8h×5d	Antibiotics standard care	28-day all-cause mortality	PCT, CRP, IL-6, TNF-a	none	?☆☆☆☆☆
Choudhuri et al. (2015)	India	2012.10-2014.05	RCT	104	68	36	P > 0.05		sepsis	NR	NR	28-day all-cause mortality	VDs, length of ICU stay, VASDs, occurrence of MODS	none	☆☆☆
Karnad et al. (2014)	India	2009.09-2010.06	RCT	114	55	59	37.5 ± 12.9	36.7 ± 12.5	sepsis	20,0000 IU q12h×5d	equivalent normal saline	28-day all-cause mortality	VDs VFDs hospital stay, APACHE II score	none	☆☆☆☆☆☆☆
Wu et al. (2013b)	China	2011.10-2012.10	RCT	60	30	30	54.3 ± 16.2		sepsis	30,0000 IU q8h×5d	equivalent normal saline	28-day all-cause mortality	MODS,IL-10,IL-6 CD4,CD25,IL-17,HLA-DR	none	☆☆☆☆☆
Sung et al. (2009)	Korea	2005.01-2008.06	PC	169	43	126	61 ± 18	61 ± 17	severe sepsis septic shock	100,0000 IU qd	Antibiotics standard care	mortality	SOFA score	none	☆☆☆
Shao et al. (2005)	China	NR	RCT	60	30	30	43.3 ± 9.2		sepsis	10,0000 IU q8h×5d	Antibiotics standard care	mortality	IL-6, IL-10, TNF-a, CRP	none	☆☆☆☆☆☆
Pavan Kumar et al. (2017)	India	2014.10-2017.10	PO	225	87	138	P > 0.05		sepsis	20,0000 IU q12h×5d	Antibiotics standard care	all-cause mortality	VFDs VASFDs	none	☆☆☆
Chen et al. (2015)	China	2013.07-2014.06	RCT	50	25	25	43.6 ± 5.8	41.7 ± 3.8	severe sepsis	20,0000 IU q12h×7 d	equivalent normal saline	28-day all-cause mortality	IL-8,TNF-α,IL-6,IL-10	none	☆☆☆☆
Tang et al. (2013)	China	NR	RCT	74	37	37	31-52		severe sepsis	20,0000 IU q12h×7d	equivalent normal saline	all-cause mortality	IL-8, TNF-α,IL-6,IL-10	none	☆☆☆☆
Jiang et al. (2006)	China	2001.12-2005.12	RCT	78	39	39	56 ± 21	54 ± 16	Severe sepsis septic shock	20,0000 IU qd×3d	equivalent normal saline	NR	IL-8,IL-1, TNF-α,IL-6	none	☆☆☆☆
Ni et al. (2008)	China	2006.1-2007.2	RCT	42	21	21	60.18 ± 19.08	59.39 ± 21.11	severe sepsis	10000 IU/kg/d q12h×5d	equivalent normal saline	28-day mortality	IL-10, TNF-α, APACHE II score	none	☆☆☆☆☆
Fang et al. (2005)	China	2003.09-2004.02	RCT	56	28	28	57 ± 16	61 ± 16	sepsis	20,0000 IU q12h×5d	equivalent normal saline	28-day mortality	IL-8, TNF-α,IL-6, APACHE II score	rash	☆☆☆☆
Dai et al. (2016)	China	2013.07-2014.06	RCT	86	43	43	59.45 ± 6.54	59.32 ± 6.15	severe sepsis	20,0000 IU q12h×5d	equivalent normal saline	NR	IL-8, TNF-α,IL-10, APACHE II score	nausea, fatigue and rash	☆☆☆☆☆
Wang et al. (2007)	China	2004.1-2006.12	RCT	84	44	40	55.3 ± 24.5	52.1 ± 16.3	sepsis	20,0000 IU q12h×7d	equivalent normal saline	NR	TNF-α,IL-6,IL-10,IL-8,IL-1	none	☆☆☆☆
Wu et al. (2016)	China	2011-2012	RCT	60	31	29	48.71 ± 30.15	50.09 ± 29.11	sepsis	20,0000 IU q8h×8d	equivalent normal saline	28-day mortality	TNF-α,IL-10 APACHE II score	None	☆☆☆☆

RCT, Randomized controlled study; PC, prospective case–control study, PO, prospective observational study; p > 0.05, no difference in the baseline of mean age; VFDs, ventilator-free days; VDs, ventilator days; VASFDs, vasopressor-free days; VASDs, vasopressor days; NR, It was not given in the original article.

### Quality Assessment

First, two reviewers independently assessed the eligibility of articles identified during the initial search strategy. Then, the quality of all included studies was evaluated according to the modified Jadad scale, which can intuitively assess the quality of the included RCTs (Jadad et al., 1996). The studies were rated as low quality and high quality under scores of 1–3 and 4–7, respectively. In this meta-analysis, three studies were regarded as low quality and 12 studies were regarded as high quality. Moreover, detailed scoring results are shown in **Table 3**.

**Table 3:** The modified Jadad questionnaire for the included studies.

Study, year (Station)	Random sequence production			Allocation concealment				Blinding metod			Withdrawals and dropouts		Score
	Adequate 2’	Unclear 1’	Inadequate 0’	Adequate 2’	Unclear 1’	Inadequate 0’	Unused 0’	Adequate 2’	Unclear 1’	Inadequate 0’	Description 1’	Undescribed 0’	
Wang et al., 2007		1			1			2				0	4
Jiang et al. (2006)		1			1			2				0	4
Karnad et al., 2014		2			2			2			1		7
Wu et al., 2013b		2			1			2				0	5
Sung et al., 2009		1			1				1			0	3
Wu et al., 2016		1			1			2				0	4
Dai et al. (2016)		2			1			2				0	5
Tang et al. (2013)		1			1			2				0	4
Chen et al., 2015		1			1			2				0	4
Fang et al. (2005)		1			1			2				0	4
Ni et al. (2008)		2			1			2				0	5
Shao et al., 2005	2			2				2				0	6
Fang and Zhao, 2017	2			2				2				0	6
Pavan Kumar et al., 2017		1			1				1			0	3
Choudhuri et al., 2015		1			1				1			0	3

1–3 score, low quality; 4–7 score, high quality.

### Statistical Analysis

In this meta-analysis, all statistical calculations and analysis were performed using Review Manager 5.3 (Cochrane collaboration, Oxford, UK). According to the results of statistical analysis, we divided the type of data into dichotomous and continuous. For the dichotomous data, such as mortality, the incidence of MODS, and adverse events, we calculated the odds ratios (OR), 95% confidence intervals (CIs) of every included study, and the overall Mantel–Haenszel (M-H). For the continuous data, such as IL-6, IL-10, and TNF-α levels and the APACHE II score, we calculated the mean difference (MD) and 95% CIs. The statistical heterogeneity was examined using chi-square and I^2^ statistical tests as well as P values. At first, we used a fixed-effects model, but then chose to employ the random-effects model if I^2^ was ≥50%.

Because patients with sepsis have a high mortality rate and not all studies report a 28-day mortality rate, we chose all-cause mortality to be our primary outcome. Sensitivity analysis was used to judge whether the study results were statistically significant. For eliminating publication bias, we used the funnel plot method.

## Results

### Description of Eligible Studies

We identified 15 (Fang and Chen, 2005; Jiang et al., 2006; Wang et al., 2007; Ni et al., 2008; Sung et al., 2009; Tang, 2013; Wu et al., 2013b; Karnad et al., 2014; Chen et al., 2015; Dai and Wang, 2016; Wu et al., 2016; Fang and Zhao, 2017; Choudhuri et al., 2015; Shao et al., 2005; Pavan Kumar et al., 2017) potential studies that included a total of 1358 patients: 630 patients in the UTI group and 728 patients in the control group. Thirteen RCTs and two prospective studies were included in this meta-analysis. The specific method for identifying studies and establishing the inclusion and exclusion criteria is shown in **Figure 1**. Eleven studies were published from China, three from India, and one from Korea.

### Primary Outcomes

#### All-Cause Mortality

**Figure 2:**
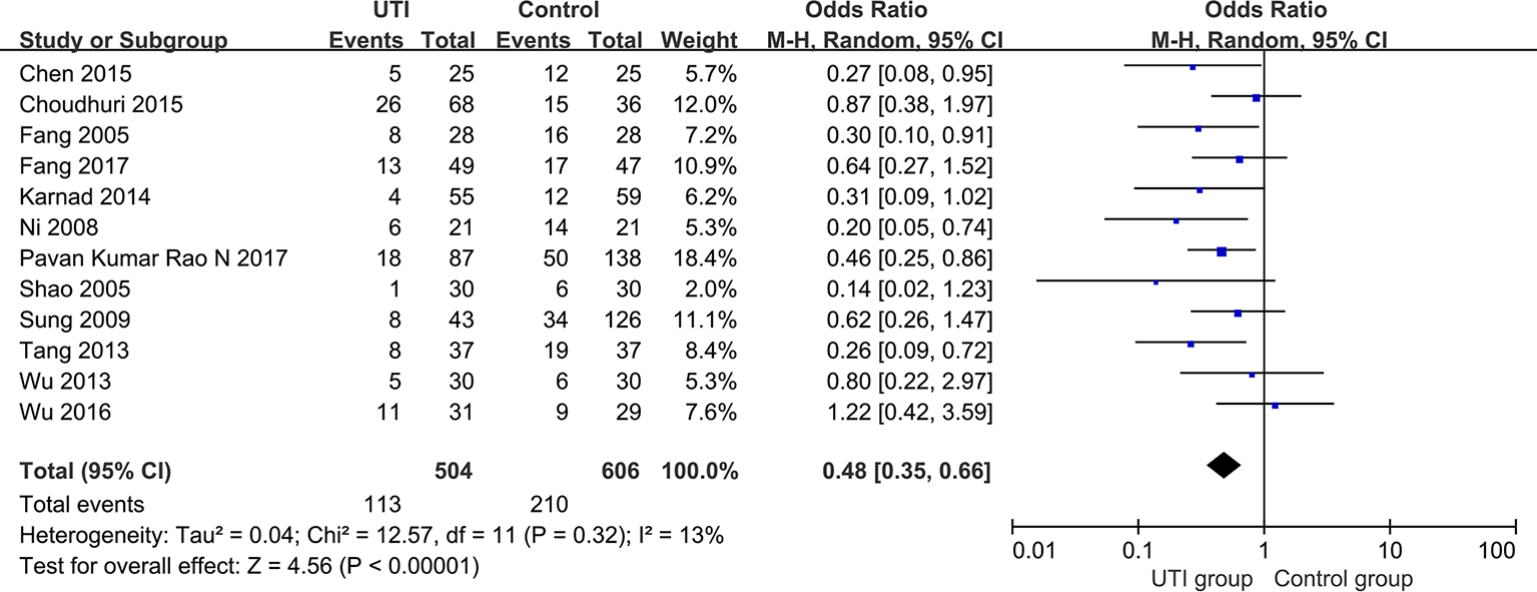
All-cause mortality of the included studies.

We extracted the data from 12 studies (Fang and Chen, 2005; Shao et al., 2005; Ni et al., 2008; Sung et al., 2009; Tang, 2013; Wu et al., 2013b; Karnad et al., 2014; Chen et al., 2015; Choudhuri et al., 2015; Wu et al., 2016; Fang and Zhao, 2017; Pavan Kumar Rao et al., 2017) and 1110 participants were classified into two groups to assess all-cause mortality. All-cause mortality was significantly lower in the UTI group than in the control group (OR = 0.48, 95% CI [0.35, 0.66], p < 0.00001), and heterogeneity was low (x^2^ = 12.57, p = 0.32, I^2^ = 13%). The results are shown in **Figure 2**.

### Secondary Outcomes

#### Levels of IL-6

**Figure 3:**
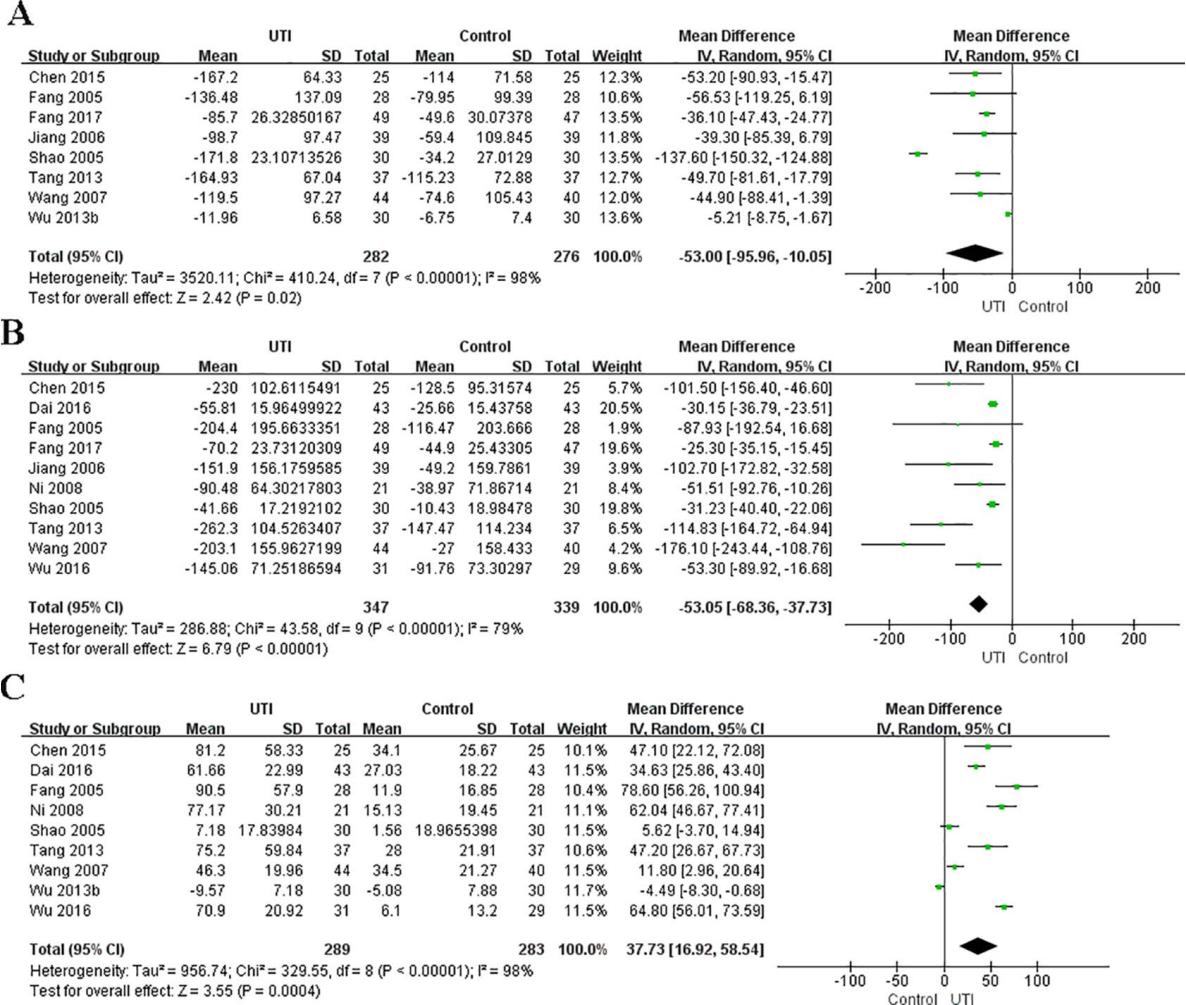
Ulinastatin regulates the levels of pro-inflammatory and anti-inflammatory cytokines. Pro-inflammatory factor: IL-6 **(A)**, TNF-α **(B)**. Anti-inflammatory factors: IL-10 **(C)**.

We obtained the related data from eight studies (Fang and Chen, 2005; Shao et al., 2005; Jiang et al., 2006; Wang et al., 2007; Tang, 2013; Wu et al., 2013b; Chen et al., 2015; Fang and Zhao, 2017) (558 participants in two groups) to analyze the serum levels of IL-6. The serum level of IL-6 at the time of hospital admission was not different between the UTI and control groups. After treatment, IL-6 was significantly less in the UTI group than in the control group (MD = −53.00, 95% CI [−95.56,−10.05], p = 0.02), and a obvious heterogeneity in the results was observed (x^2^ = 410.24, p < 0.00001, I^2^ = 98%). The results are shown in **Figure 3A**.

#### Levels of TNF-α

We collected the related data from ten studies (Fang and Chen, 2005; Shao et al., 2005; Jiang et al., 2006; Wang et al., 2007; Ni et al., 2008; Tang, 2013; Chen et al., 2015; Dai and Wang, 2016; Wu et al., 2016; Fang and Zhao, 2017) (686 participants in two groups) to analyze the serum levels of TNF-α. The level of TNF-α at the time of hospital admission was not different between the UTI and control groups. After treatment, TNF-α was significantly less in the UTI group than in the control group (MD = −53.05, 95% CI [−68.36, −37.73], p < 0.00001), and an obvious heterogeneity was observed in the results (x^2^ = 43.58, p < 0.00001, I^2^ = 79%). The results are shown in **Figure 3B**.

#### Levels of IL-10

We gained the related data from nine studies (Fang and Chen, 2005; Shao et al., 2005; Wang et al., 2007; Ni et al., 2008; Tang, 2013; Wu et al., 2013b; Chen et al., 2015; Dai and Wang, 2016; Wu et al., 2016) (572 participants in two groups) to analyze the serum levels of IL-10. The serum level of IL-10 at the time of hospital admission was not different between the UTI and control groups. After treatment, IL-10 was significantly greater in the UTI group than in the control group (MD = 37.73, 95% CI [16.92, 58.54], p = 0.0004), and an obvious heterogeneity was observed in the results (x^2^ = 329.55, p < 0.0001, I^2^ = 98%). The results are shown in **Figure 3C**.

### The Apache II Score

**Figure 4:**
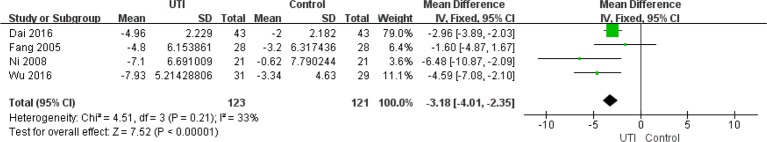
Ulinastatin reduces the APACHE II score of sepsis patients.

The APACHE II scores at the time of hospital admission were not different between the UTI and control groups. We extracted the data from four studies (Ni et al., 2008; Fang et al., 2005; Dai and Wang, 2016; Wu et al., 2016) (244 participants in two groups) to assess this change. After treatment, the APACHE II scores were significantly less in the UTI group than in the control group MD = −3.18, 95%CI [−4.01, −2.35], p < 0.00001), and heterogeneity was low (x^2^ = 4.51, p = 0.21, I^2^ = 33%). The results are shown in **Figure 4**.

### The Incidence of MODS

**Figure 5:**
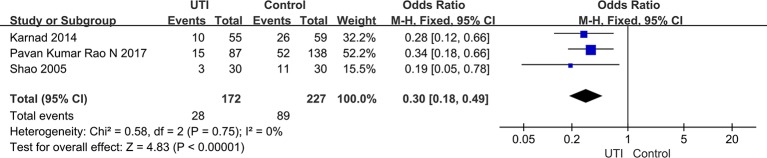
Ulinastatin reduces the incidence of multiple organ dysfunction syndrome (MODS).

We extracted the data from three studies (Shao et al., 2005; Karnad et al., 2014; Pavan Kumar Rao et al., 2017) (399 participants in included in two groups) to assess the incidence of MODS. After treatment, the incidence of MODS was significantly less in the UTI groups than in the control groups (OR = 0.3, 95% CI [0.18–0.49], p < 0.00001), and heterogeneity was not observed in the results (x^2^ = 0.58, p = 0.75, I^2^ = 0%). The result is shown in **Figure 5**.

### Publication Bias and Sensitivity Analysis

**Figure 6:**
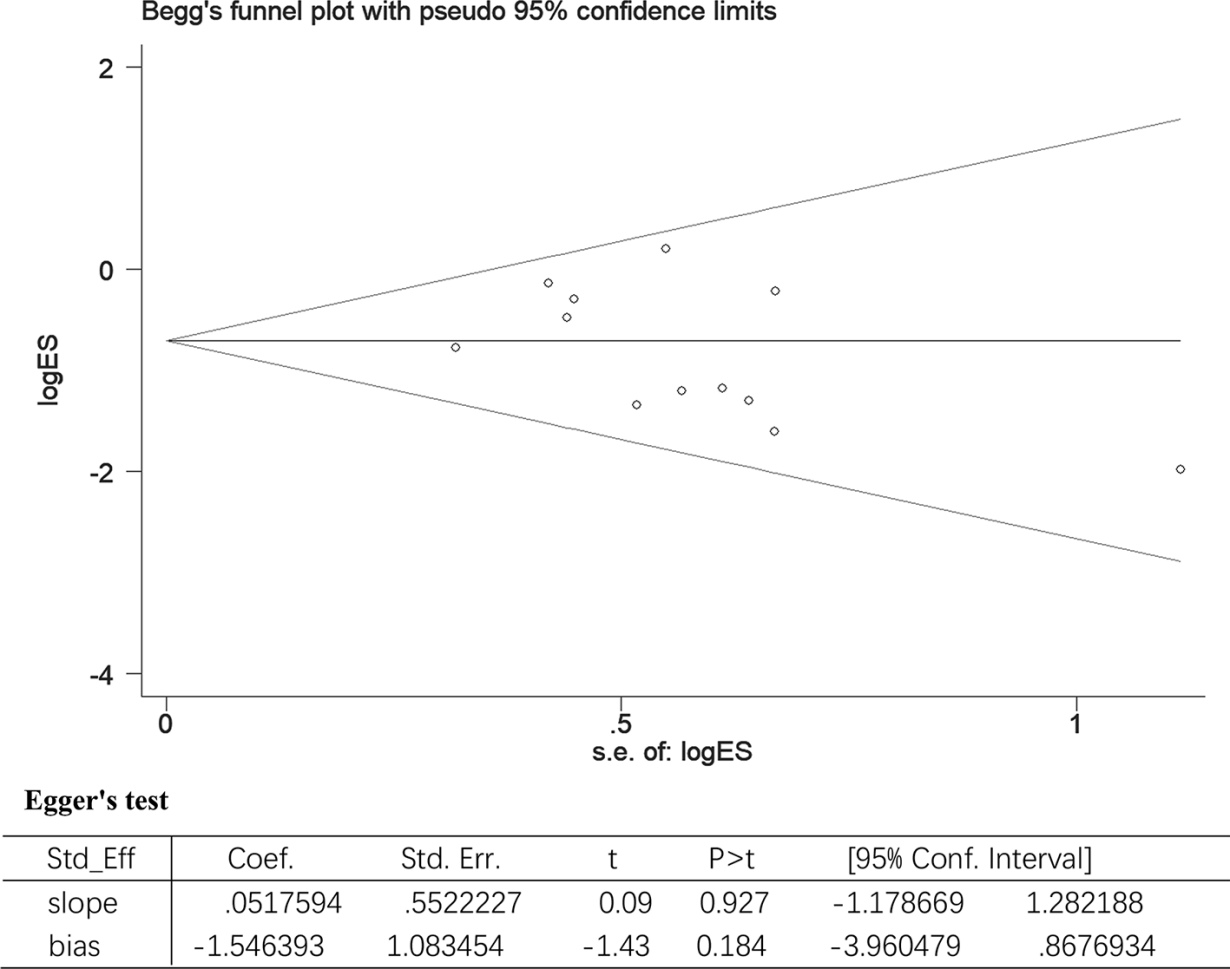
The Begg’s test and Egger’s test for all-cause-mortality. Begg’s test: rank correlation test; Egger’s test: linear regression method; ES, effect size; 95% CI, 95% confidence interval.

All included studies involved the use of UTI for treating sepsis patients. Using all-cause mortality as the main variable, the included studies were evaluated for the effect of study size. The funnel plot demonstrated a balanced and or a symmetrical shape, suggesting no significant publication bias. In addition, Egger’s test also demonstrated a statistically significant symmetry (p = 0.183). Therefore, the potential publication bias had no significant influence on the results (**Figure 6**).

Sensitivity analysis was conducted by the leave-one-out method and checking the consistency of the overall effect estimate. For IL-6, we found that the I^2^ value decreased to 0% after excluding the studies conducted by Shao et al. (2005) and Wu et al. (2013b). For TNF-α, we found that the I^2^ value decreased to 54% after excluding the studies conducted by Tang et al. (2013) and Wang et al. (2007). For IL-10, we found that the I^2^ value decreased up to 84% after removing the study by Shao et al. (2005), Wang et al. (2007) and Wu et al. (2013b). We believe that the high heterogeneity may arise from factors such as sample size, different measuring instruments, and design methods.

## Discussion

UTI is a multifunctional Kunitz-type serine protease inhibitor found in human urine and blood. UTI is a member of inter-a-inhibitor (IaI) family, which is produced by hepatocytes (Linder and Russell, 2014). It was originally used to treat acute pancreatitis or hyperthermia (Itaba et al., 2013; Zhang et al., 2016). Subsequently, more studies revealed its use in anti-inflammation and the protection of liver function as well as in cardiopulmonary bypass and lung disease treatment (Song et al., 2011; Li et al., 2016). In such conditions, UTI can inhibit the inflammatory response, scavenge oxygen free radicals, and shorten the time of tracheal intubation and ventilation (Yang et al., 2011; Hui et al., 2014). At present, UTI has been evidenced to provide an attractive “rescue” therapeutic option for endotoxin-related inflammatory disorders such as disseminated intravascular coagulation (DIC), acute lung injury, and acute liver injury (Inoue and Takano, 2010). Recently, UTI has been demonstrated to play a vital role in sepsis. It is well known that the immune state of sepsis patients undergoes complex changes from the onset of hyper-inflammatory response in the early phase to the immune paralysis in the late phase. To date, no drug has been specifically approved to treat sepsis in human. Recent studies show that UTI has the capacity to reduce inflammation and protect cells and has a potential survival benefit in sepsis and MODS (Linder and Russell, 2014; Atal and Atal, 2016; Chang et al., 2017a). In the past, the research on UTI treatment for sepsis has mainly been conducted in China, and the results suggested UTI administration can reduce sepsis patient mortality. In 2014, a randomized, controlled, double-blind, and multi-center trial was conducted in India, which revealed the survival benefit of UTI in patients with sepsis (Karnad et al., 2014). A meta-analysis of the effects of UTI combined with thymosin α1 revealed a reduction in mortality. To better clarify the efficacy of UTI or thymosin α1 administration on sepsis patients, we pooled the RCTs involving treatment with UTI alone. In this meta-analysis, 13 relevant RCTs from three countries and two prospective studies were included. The results showed that in patients with sepsis or septic shock UTI was associated with a significant decrease in all-cause mortality and improvements in both inflammatory cytokine profiles and APACHE II scores. Mortality is the most important index for efficacy evaluation. Studies conducted in sepsis models support that UTI is capable of reducing sepsis-related mortality. The survival benefits were also observed in most clinical trials conducted in different countries. Several trials failed to achieve positive results, probably owing to an insufficient sample size and differences in patients, trials design, and other clinical factors.

Anti-inflammation is one of the most important properties of UTI. It is well known that systemic inflammatory response plays a key role in organ damage or death in sepsis. Agents directed at a single inflammatory mediator have not been shown to have a protective effect in sepsis patients. These results suggest that a single anti-inflammatory agent cannot disrupt the complicated inflammatory network. However, the removal of blood mediators by continuous renal replacement therapy (CRRT) facilitated the achievement of a survival benefit in patients with sepsis or septic shock (Liu et al., 2011; Servillo et al., 2013). Similarly with CRRT, UTI demonstrated a capacity to decrease diverse inflammatory mediator factors such as IL-1, IL-8, IL-6, HMGB1, and other mediators. UTI also inhibits inflammation by suppressing the infiltration of neutrophils and release of elastase and inflammatory mediators from neutrophils. UTI can also suppress MAPK-signaling pathway, which mediates the release of inflammatory cytokines such as TNF-α, IL-1, and IL-6 (Inoue and Takano, 2010; Fang et al., 2018). Recently, a retrospective study of 263 critically ill patients with sepsis found that 28-day mortality decreased significantly with UTI (Xu et al., 2018). The authors concluded that 35% of the total effect of UTI was associated with the reduction in C-reactive protein (CRP), a major marker of inflammation.

Anti-apoptosis is another property of UTI. It has been observed that UTI can reduce apoptosis of endothelial cells, lymphocytes, intestinal epithelium, neurons, and renal cells during different diseases and in animal models (Li et al., 2014). It is well known that there are many immune cells, including lymphocytes, monocytes, and dendritic cells that undergo apoptosis during sepsis. It has been confirmed that UTI can protect cells from apoptosis though anti-oxidation and reduction of mitochondrial damage. It is known that apoptosis contributes to immunoparalysis and death of sepsis patients (Hotchkiss and Nicholson, 2006; Hotchkiss et al., 2013; Chang et al., 2017b). Anti-apoptosis in sepsis models, *via* increase in Bcl-2 expression or blocking of CD95, reduced the incidence of sepsis-related mortality (Hotchkiss and Nicholson, 2006; Zhang et al., 2010; Sun et al., 2011; Liu et al., 2013). In clinical trials, anti-immune cell apoptosis with anti-PD-1 or anti-PD-L1 also showed potential in sepsis treatment (Zhang et al., 2010; Patera et al., 2016). These studies suggest that cell protection may also be involved in UTI-related survival benefit in patients with sepsis.

## Limitations

Although this meta-analysis reveals the potential benefits of UTI inpatients with sepsis, these trials were conducted mainly in single centers and the sample sizes were small. Recently, a retrospective observational study conducted in a single intensive care unit (ICU) by Uchida et al. (Uchida et al., 2018) found that UTI was not associated with a mortality benefit in elderly patients with established multiple organ failure from a variety of causes, only a minority of which were sepsis related. However, UTI use was associated with reduced time on both mechanical ventilators and vasoactive drugs. Thus, multicenter, large sample, randomized clinical trials are still urgently needed to further evaluate the effects of UTI in patients with sepsis. At present, ADJunctive Ulinastatin in Sepsis Treatment in China (ADJUST study), a large sample, multi-center, double-blind, randomized, parallel-group, placebo-controlled trial is being conducted in mainland China (Jiang et al., 2018). The aim of this trial is to further evaluate the efficacy and safety profiles of UTI.

## Conclusions

UTI is associated with reductions in both all-cause mortality and the incidence of MODS, and improvements in both APACHE II scores and inflammatory cytokine profiles in patients with sepsis, severe sepsis, or septic shock. Large high quality RCTs are needed to confirm these promising results of UTI in sepsis and septic shock.

## Data Availability Statement

The data analyzed in this study was obtained from PubMed, Medline, Embase, and China National Knowledge Infrastructure (CNKI), the following licenses apply. Requests to access these datasets should be directed to HW, 970092671@qq.com.

## Author Contributions

ZL conceived and designed the study. HW, LY, YT, BH, ZL, and PC conducted the literature search, read initial abstracts, extracted data from potential eligible studies, and conducted the statistical analyses. HW and BL wrote the first draft of the manuscript. ZL, PC, RL, and BL contributed with manuscript writing, concrete suggestions, and manuscript revision.

## Funding

This work is supported by Clinical Research Startup Program of Southern Medical University by High-level University Construction Funding of Guangdong Provincial Department of Education(LC2019ZD014).

## Conflict of Interest

The authors declare that the research was conducted in the absence of any commercial or financial relationships that could be construed as a potential conflict of interest.

## References

[B1] Angus D. C. Linde-Zwirble W. T. Lidicker J. Clermont G. Carcillo J. Pinsky M. R. (2001). Epidemiology of severe sepsis in the United States: analysis of incidence, outcome, and associated costs of care. Crit. Care Med. 29 (7), 1303–1310. 10.1097/00003246-200107000-00002 11445675

[B2] Armstrong B. A. Betzold R. D. May A. K. (2017). Sepsis and septic shock strategies. Surg. Clin. North Am. 97 (6), 1339–1379. 10.1016/j.suc.2017.07.003 29132513

[B3] Atal S. S. Atal S. (2016). Ulinastatin - a newer potential therapeutic option for multiple organ dysfunction syndrome. J. Basic Clin. Physiol. Pharmacol. 27 (2), 91–99. 10.1515/jbcpp-2015-0003 26565549

[B4] Chang P. Liu J. Yu Y. Cui S. Y. Guo Z. H. Chen G. M. (2017a). Alpha-lipoic acid suppresses extracellular histone-induced release of the infammatory mediator tumor necrosis factor-alpha by macrophages. Cell Physiol. Biochem. 42 (6), 2559–2568. 10.1159/000480217 28848097

[B5] Chang P. Mo B. Cauvi D. M. Yu Y. Guo Z. Zhou J. (2017b). Grape seed proanthocyanidin extract protects lymphocytes against histone-induced apoptosis. PeerJ 5, e3108. 10.7717/peerj.3108 28344907 PMC5363264

[B6] Chen W. Peng S. Chen Y. (2015). Comparative observation of curative effects by ulinastatin and conventional method in the treatment of severe sepsis. Chinese J. Modern Drug App. 9 (7), 1–2, 3. 10.14164/j.cnki.cn11-5581/r.2015.07.001

[B7] Choudhuri A. H. Tyagi R. Tyagi R. Agarwal D. Uppal R. (2015). Early use of ulinastatin reduces Multiorgan Dysfunction (MODS) in septic shock following anastomotic failure. Clin. Therapeutics 37 (8), e112.

[B8] Chousterman B. G. Swirski F. K. Weber G. F. (2017). Cytokine storm and sepsis disease pathogenesis. Semin Immunopathol. 39 (5), 517–528. 10.1007/s00281-017-0639-8 28555385

[B9] Dai Q. X. Wang C. H. (2016). Effects of urinary trypsin inhibitor on serum levels of TNF-a, IL-8 and IL-10 in patients with severe sepsis. Chinese J. Biochem. Pharmaceutics 36 (3), 146–148. 10.3969/j.issn.1005-1678.2016.03.48

[B10] Fang Q. Chen P. (2005). A Study on clinical efficacy of ulinastatin in severe sepsis patients and its mechanism of action. Chinese J. Of Infect. And Chemother. 5 (1), 13–16. 10.3321/j.issn:1009-7708.2005.01.003

[B11] Fang Q. Zhao X. (2017). Clinical effect of combined ulinastatin and continuous renal replacement therapy on management of severe sepsis with acute kidney injury. Tropical J. Pharmaceutical Res. 16 (4), 925–930. 10.4314/tjpr.v16i4.26

[B12] Fang M. Zhong W.-H. Song W.-L. Deng Y.-Y. Yang D.-M. Xiong B. (2018). Ulinastatin ameliorates pulmonary capillary endothelial permeability induced by sepsis through protection of tight junctions via inhibition of TNF-alpha and related pathways. Front. Pharmacol. 9, 823. 10.3389/fphar.2018.00823 30150933 PMC6099086

[B13] Feng Z. Shi Q. Fan Y. Wang Q. Yin W. (2016). Ulinastatin and/or thymosin alpha1 for severe sepsis: a systematic review and meta-analysis. J. Trauma Acute Care Surg. 80 (2), 335–340. 10.1097/ta.0000000000000909 26517783

[B14] Girardot T. Rimmele T. Venet F. Monneret G. (2017). Apoptosis-induced lymphopenia in sepsis and other severe injuries. Apoptosis 22 (2), 295–305. 10.1007/s10495-016-1325-3 27812767

[B15] Han D. Shang W. Wang G. Sun L. Zhang Y. Wen H. (2015). Ulinastatin- and thymosin alpha1-based immunomodulatory strategy for sepsis: a meta-analysis. Int. Immunopharmacol. 29 (2), 377–382. 10.1016/j.intimp.2015.10.026 26522590

[B16] Harjai M. Bogra J. Kohli M. Pant A. B. (2013). Is suppression of apoptosis a new therapeutic target in sepsis?. Anaesth. Intensive Care 41 (2), 175–183. 10.1177/0310057X1304100207 23530784

[B17] Hotchkiss R. S. Crouser E. (2015). Imaging apoptosis in sepsis–A technology we would die for!. Crit. Care Med. 43 (11), 2506–2508. 10.1097/ccm.0000000000001289 26468702 PMC4837329

[B18] Hotchkiss R. S. Nicholson D. W. (2006). Apoptosis and caspases regulate death and inflammation in sepsis. Nat. Rev. Immunol. 6 (11), 813–822. 10.1038/nri1943 17039247

[B19] Hotchkiss R. S. Monneret G. Payen D. (2013). Sepsis-induced immunosuppression: from cellular dysfunctions to immunotherapy. Nat. Rev. Immunol. 13 (12), 862–874. 10.1038/nri3552 24232462 PMC4077177

[B20] Hui L. Shen F. Chang H. Li X. Gao G. Ma J. (2014). Effects of ulinastatin on cerebral oxygen metabolism and CRP levels in patients with severe traumatic brain injury. Exp. Therapeutic Med. 7 (6), 1683–1686. 10.3892/etm.2014.1666 PMC4043583 24926366

[B21] Inoue K.-I. Takano H. (2010). Urinary trypsin inhibitor as a therapeutic option for endotoxin-related inflammatory disorders. Expert Opin. Investig. Drugs 19 (4), 513–520. 10.1517/13543781003649533 20367192

[B22] Itaba S. Nakamura K. Aso A. Tokunaga S. Akiho H. Ihara E. (2013). Prospective, randomized, double-blind, placebo-controlled trial of ulinastatin for prevention of hyperenzymemia after double balloon endoscopy via the antegrade approach. Dig. Endosc. 25 (4), 421–427. 10.1111/den.12014 23368820

[B23] Jadad A. R. Moore R. A. Carroll D. Jenkinson C. Reynolds D. J. Gavaghan D. J. (1996). Assessing the quality of reports of randomized clinical trials: is blinding necessary?. Control Clin. Trials 17 (1), 1–12. 10.1016/0197-2456(95)00134-4 8721797

[B24] Jiang L. Y. Hong Y. L. Xing C. J. (2006). The effect of Ulinastatin on the delivery of cytokines in patients with septic shock. Chinese J. Of Emergency Med. 15 (1671-0282), 1136–1138. 10.3760/j.issn:1671-0282.2006.12.020

[B25] Jiang W. Yu X. Sun T. Chai Y. Chang P. Chen Z. (2018). ADJunctive Ulinastatin in Sepsis Treatment in China (ADJUST study): study protocol for a randomized controlled trial. Trials 19 (1), 133. 10.1186/s13063-018-2513-y 29467017 PMC5822617

[B26] Karnad D. R. Bhadade R. Verma P. K. Moulick N. D. Daga M. K. Chafekar N. D. (2014). Intravenous administration of ulinastatin (human urinary trypsin inhibitor) in severe sepsis: a multicenter randomized controlled study. Intensive Care Med. 40 (6), 830–838. 10.1007/s00134-014-3278-8 24737258 PMC4028549

[B27] Li G. Li T. Li Y. Cai S. Zhang Z. Zeng Z. (2014). Ulinastatin inhibits oxidant-induced endothelial hyperpermeability and apoptotic signaling. Int. J. Clin. Exp. Pathol. 7 (11), 7342–7350. 25550770 PMC4270631

[B28] Li C. Bo L. Liu Q. Jin F. (2015). Thymosin alpha1 based immunomodulatory therapy for sepsis: a systematic review and meta-analysis. Int. J. Infect. Dis. 33, 90–96. 10.1016/j.ijid.2014.12.032 25532482

[B29] Li C. Ma D. Chen M. Zhang L. Zhang L. Zhang J. (2016). Ulinastatin attenuates LPS-induced human endothelial cells oxidative damage through suppressing JNK/c-Jun signaling pathway. Biochem. Biophys. Res. Commun. 474 (3), 572–578. 10.1016/j.bbrc.2016.04.104 27109479

[B30] Liberati A. Altman D. G. Tetzlaff J. Mulrow C. Gotzsche P. C. Ioannidis J. P. (2009). The PRISMA statement for reporting systematic reviews and meta-analyses of studies that evaluate healthcare interventions: explanation and elaboration. BMJ 339, b2700. 10.1136/bmj.b2700 19622552 PMC2714672

[B31] Linder A. Russell J. A. (2014). An exciting candidate therapy for sepsis: ulinastatin, a urinary protease inhibitor. Intensive Care Med. 40 (8), 1164–1167. 10.1007/s00134-014-3366-9 24990495

[B32] Liu Z.-G. Zhang J. He Y. Chang P. Gu D.-S. Luo Y.-W. (2011). Impact of transfusion of apoptotic and necrotic thymocytes on the survival of mice with sepsis. J. Of Southern Med. Univ. 31 (6), 960–964. 21690045

[B33] Liu Z. G. Ni S. Y. Chen G. M. Cai J. Guo Z. H. Chang P. (2013). Histones-mediated lymphocyte apoptosis during sepsis is dependent on p38 phosphorylation and mitochondrial permeability transition. PLoS One 8 (10), e77131. 10.1371/journal.pone.0077131 24167561 PMC3805602

[B34] Liu D. Yu Z. Yin J. Chen Y. Zhang H. Fan X. (2017). Effect of ulinastatin combined with thymosin alpha1 on sepsis: a systematic review and meta-analysis of Chinese and Indian patients. J. Crit. Care 39, 285–287. 10.1016/j.jcrc.2017.02.005 28283220

[B35] Martin G. S. Mannino D. M. Eaton S. Moss M. (2003). The epidemiology of sepsis in the United States from 1979 through 2000. N. Engl. J. Med. 348 (16), 1546–1554. 10.1056/NEJMoa022139 12700374

[B36] Minasyan H. (2017). Sepsis and septic shock: Pathogenesis and treatment perspectives. J. Crit. Care 40, 229–242. 10.1016/j.jcrc.2017.04.015 28448952

[B37] Ni H. Y. Fang Q. Zhang Y. S. (2008). Effects of ulinastatin on Inflammatory response and curative effect in severe sepsis patients. Chinese J. Of Crit. Care Med. 28 (4), 342–344. 10.3969/j.issn.1002-1949.2008.04.017

[B38] Patera A. C. Drewry A. M. Chang K. Beiter E. R. Osborne D. Hotchkiss R. S. (2016). Frontline Science: defects in immune function in patients with sepsis are associated with PD-1 or PD-L1 expression and can be restored by antibodies targeting PD-1 or PD-L1. J. Leukoc. Biol. 100 (6), 1239–1254. 10.1189/jlb.4HI0616-255R 27671246 PMC5110001

[B39] Pavan Kumar Rao N. Sv S. Nayak K. S. (2017). Use of ulinastatin in renal failure patients developing sepsis. Indian J. Nephrol. 27, S68–S69.

[B40] Rajaee A. Barnett R. Cheadle W. G. (2018). Pathogen- and danger-associated molecular patterns and the cytokine response in sepsis. Surg. Infect. (Larchmt) 19 (2), 107–116. 10.1089/sur.2017.264 29364781

[B41] Servillo G. Vargas M. Pastore A. Procino A. Iannuzzi M. Capuano A. (2013). Immunomodulatory effect of continuous venovenous hemofiltration during sepsis: preliminary data. Biomed. Res. Int. 2013, 108951. 10.1155/2013/108951 23971020 PMC3736510

[B42] Shao Y. M. Zhang L. Q. Deng L. H. Yao H. G. (2005). [Clinical study on effects of ulinastatin on patients with systemic inflammatory response syndrome]. Zhongguo Wei Zhong Bing Ji Jiu Yi Xue 17 (4), 228–230. 15836828

[B43] Shu H. Liu K. He Q. Zhong F. Yang L. Li Q. (2014). Ulinastatin, a protease inhibitor, may inhibit allogeneic blood transfusion-associated pro-inflammatory cytokines and systemic inflammatory response syndrome and improve postoperative recovery. Blood Transfus. 12 Suppl 1, s109–s118. 10.2450/2013.0224-12 23736923 PMC3934215

[B44] Singer M. Deutschman C. S. Seymour C. W. Shankar-Hari M. Annane D. Bauer M. (2016). The third international consensus definitions for sepsis and septic shock (Sepsis-3). Jama 315 (8), 801–810. 10.1001/jama.2016.0287 26903338 PMC4968574

[B45] Song J. E. Kang W. S. Kim D. K. Yoon T. G. Kim T. Y. Bang Y. S. (2011). The effect of ulinastatin on postoperative blood loss in patients undergoing open heart surgery with cardiopulmonary bypass. J. Int. Med. Res. 39 (4), 1201–1210. 10.1177/147323001103900408 21986122

[B46] Stoller J. Halpin L. Weis M. Aplin B. Qu W. Georgescu C. (2016). Epidemiology of severe sepsis: 2008-2012. J. Crit. Care 31 (1), 58–62. 10.1016/j.jcrc.2015.09.034 26601855

[B47] Sun R. Zhang Y. Lv Q. Liu B. Jin M. Zhang W. (2011). Toll-like receptor 3 (TLR3) induces apoptosis via death receptors and mitochondria by up-regulating the transactivating p63 isoform alpha (TAP63alpha). J. Biol. Chem. 286 (18), 15918–15928. 10.1074/jbc.M110.178798 21367858 PMC3091201

[B48] Sung W. M. Sung W. L. Yun S. H. Dae W. P. Ik J. J. Young H. Y. (2009). The effects of urinary trypsin inhibitor on the outcomes of severe sepsis and septic shock patients. Korean Emergency Medical J. 20, 80–85.

[B49] Tang J. (2013). The clinical effect analysis of treating septicopyemia patients with ulinastatin. Guide China Med. (15), 439–440. 10.3969/j.issn.1671-8194.2013.15.333

[B50] Uchida M. Abe T. Ono K. Tamiya N. (2018). Ulinastatin did not reduce mortality in elderly multiple organ failure patients: a retrospective observational study in a single center ICU. Acute Med. Surg. 5 (1), 90–97. 10.1002/ams2.304 29445506 PMC5797838

[B51] Wang Y. Li X. Kexi L. (2007). Influence of Ulinastatin on cytokines of patients with sepsis. Modern Med. J. Of China 9 (11), 23–25. 10.3969/j.issn.1672-9463.2007.11.008

[B52] Wang F. Y. Fang B. Qiang X. H. Yu T. O. Zhong J. R. Cao J. (2016). The efficacy and immunomodulatory effects of ulinastatin and thymosin alpha1 for sepsis: a systematic review and meta-analysis. Biomed. Res. Int. 2016, 9508493. 10.1155/2016/9508493 27340674 PMC4906180

[B53] Wiedow O. Meyer-Hoffert U. (2005). Neutrophil serine proteases: potential key regulators of cell signalling during inflammation. J. Intern. Med. 257 (4), 319–328. 10.1111/j.1365-2796.2005.01476.x 15788001

[B54] Wong W. W. (1998). ICE family proteases in inflammation and apoptosis. Agents Actions Suppl 49, 5–13. 10.1007/978-3-0348-8857-8_2 9426822

[B55] Wu J. Zhou L. Liu J. Ma G. Kou Q. He Z. (2013a). The efficacy of thymosin alpha 1 for severe sepsis (ETASS): a multicenter, single-blind, randomized and controlled trial. Crit. Care 17 (1), R8. 10.1186/cc11932 23327199 PMC4056079

[B56] Wu T. J. Zhang L. N. Kang C. C. (2013b). The effect of ulinastatin on disbalance of inflammation and immune status in patients with severe sepsis. Zhonghua Wei Zhong Bing Ji Jiu Yi Xue 25 (4), 219–223. 10.3760/cma.j.issn.2095-4352.2013.04.010 23660098

[B57] Wu Y. Cui Z. B. Kang H. S. Zhao Y. Y. Liu S. H. (2016). Effect of ulinastatin on the serum levels of tumor necrosis factor α, interleukin 10,troponin I and C-reactive protein. J. Bengbu Med. College 41 (12), 1635–1638. 10.13898/j.cnki.issn.1000-2200.2016.12.031

[B58] Xu Q. Yan Q. Chen S. (2018). Ulinastatin is effective in reducing mortality for critically ill patients with sepsis: a causal mediation analysis. Sci. Rep. 8 (1), 14360. 10.1038/s41598-018-32533-9 30254204 PMC6156583

[B59] Yang H. Mao Y. Lu X. Sang X. Du S. Zhao H. (2011). The effects of urinary trypsin inhibitor on liver function and inflammatory factors in patients undergoing hepatectomy: a prospective, randomized, controlled clinical study. Am. J. Surg. 202 (2), 151–157. 10.1016/j.amjsurg.2010.07.044 21718959

[B60] Zhang Y. Zhou Y. Lou J. Li J. Bo L. Zhu K. (2010). PD-L1 blockade improves survival in experimental sepsis by inhibiting lymphocyte apoptosis and reversing monocyte dysfunction. Crit. Care 14 (6), R220. 10.1186/cc9354 21118528 PMC3220038

[B61] Zhang H. Tan C. Wang X. Kang D. Chen Y. Xiong J. (2016). Preventive effects of ulinastatin on complications related to pancreaticoduodenectomy: a Consort-prospective, randomized, double-blind, placebo-controlled trial. Med. (Baltimore) 95 (24), e3731. 10.1097/md.0000000000003731 PMC4998438 27310952

