# Corrigendum: Improvement of Sepsis Prognosis by Ulinastatin: A Systematic Review and Meta-Analysis of Randomized Controlled Trials

**DOI:** 10.3389/fphar.2019.01697

**Published:** 2020-02-21

**Authors:** Huifang Wang, Bin Liu, Ying Tang, Ping Chang, Lishuai Yao, Bo Huang, Robert F. Lodato, Zhanguo Liu

**Affiliations:** ^1^ Department of Intensive Care Unit, Zhujiang Hospital, Southern Medical University, Guangzhou, China; ^2^ Emergency Department, Zhujiang Hospital, Southern Medical University, Guangzhou, China; ^3^ Department of Thoracic and Cardiovascular Surgical, Zhujiang Hospital, Southern Medical University, Guangzhou, China; ^4^ Department of Pulmonary, Critical Care, and Sleep Medicine, Medical School, University of Texas Health Science Center at Houston, Houston, TX, United States

**Keywords:** sepsis, ulinastatin, mortality, inflammatory cytokine, immune system

In the original article, there was a mistake in **Figure 3** as published. In **Figure 3**, we found that some data of TNF-a in **Figure 3B** was copied to **Figure 3A** by mistake. We reanalyzed the data and the new **Figure 3A** was generated. In **Figure 3C**, the left graph label should be “Control” and the right label should be “UTI.” This mistake was made because the system default label was not changed when using the software. The corrected **Figure 3** appears below.

**Figure 3:**
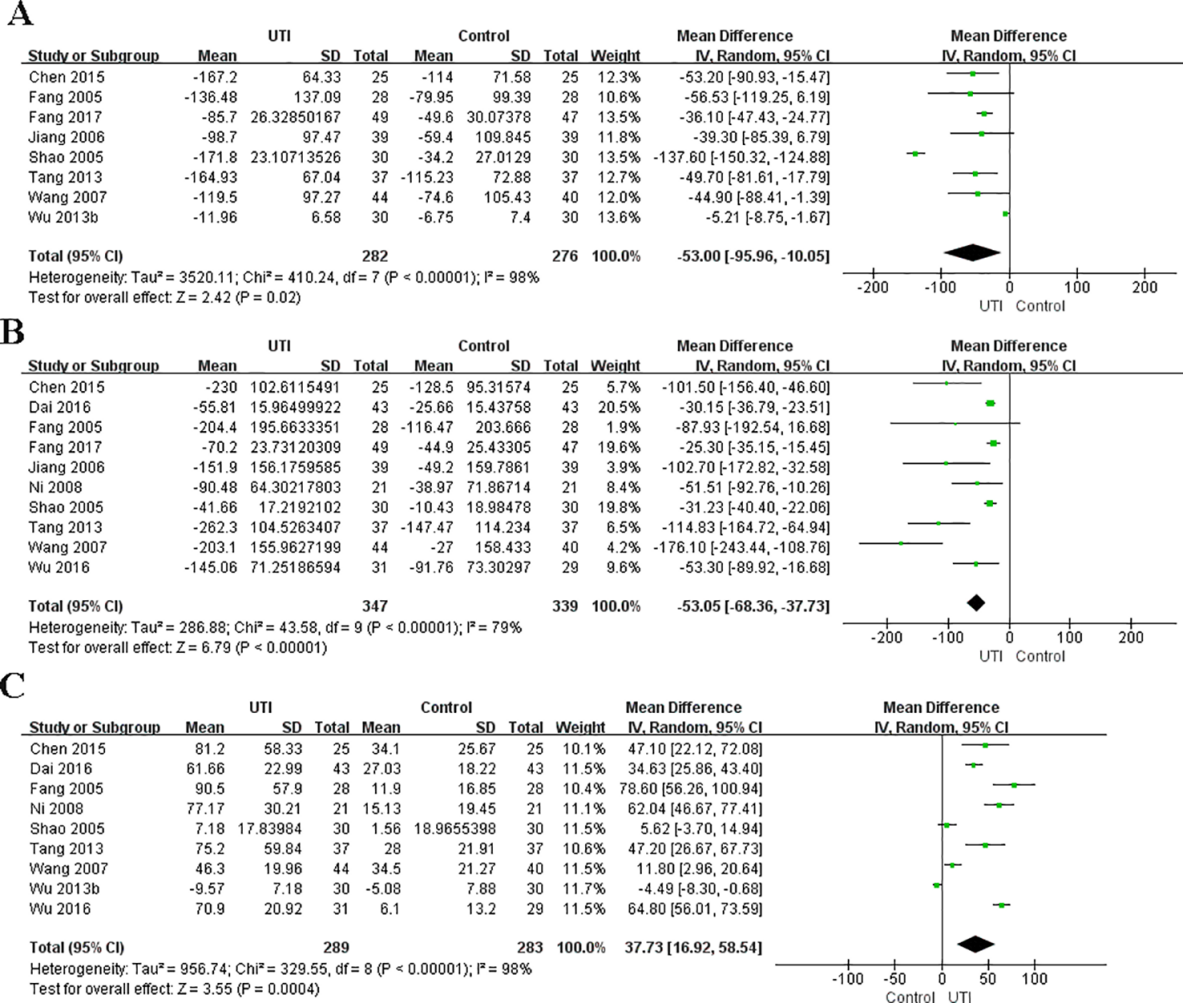
Ulinastatin regulates the levels of pro-inflammatory and anti-inflammatory cytokines. Pro-inflammatory factor: IL-6 **(A)**, TNF-α **(B)**. Anti-inflammatory factors: IL-10 **(C)**.

In **Figure 4**, Karnad et al., 2014 was mistakenly added instead of Wu et al., 2016. We reanalyzed the data and the new **Figure 4** was generated. The corrected **Figure 4** appears below.

**Figure 4:**
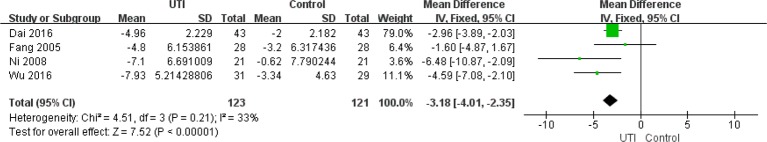
Ulinastatin reduces the APACHE II score of sepsis patients.

Additionally, Fang and Zhao, 2017; Choudhuri et al., 2015; Shao et al., 2005; Pavan Kumar et al., 2017 were not cited in the article. The citation has now been inserted in the section **Results**, subsection **Description of Eligible Studies**, paragraph 1 and should read:

“We identified 15 (Fang and Chen, 2005; Jiang et al., 2006; Wang et al., 2007; Ni et al., 2008; Sung et al., 2009; Tang, 2013; Wu et al., 2013b; Karnad et al., 2014; Chen et al., 2015; Dai and Wang, 2016; Wu et al., 2016; Fang and Zhao, 2017; Choudhuri et al., 2015; Shao et al., 2005; Pavan Kumar et al., 2017) potential studies that included a total of 1358 patients: 630 patients in the UTI group and 728 patients in the control group. Thirteen RCTs and two prospective studies were included in this meta-analysis. The specific method for identifying studies and establishing the inclusion and exclusion criteria is shown in **Figure 1**. Eleven studies were published from China, three from India, and one from Korea.”

The reference for Fang et al., 2005 was incorrectly written as Karnad et al., 2014. The citation has now been inserted in the **Results** section, subsection **The APACHE Ⅱ Score**, paragraph 1. The corrected paragraph appears below:

“The APACHE II score at the time of hospital admission were not different between the UTI and control groups. We extracted the data from four studies (Ni et al., 2008; Fang et al., 2005; Dai and Wang, 2016; Wu et al., 2016) (244 participants in two groups) to assess this change. After treatment, the APACHE II scores were significantly less in the UTI group than in the control group MD = -3.18, 95%CI [-4.01, -2.35], p < 0.00001), and heterogeneity was low (x^2^ = 4.51, p = 0.21, I^2^ = 33%). The results are shown in **Figure 4**”.

In the **Results** section, subsection **Publication Bias and Sensitivity Analysis**, paragraph 2, the first citation of Wang et al. (2007) should be Wu et al. (2013b). The corrected paragraph appears below:

“Sensitivity analysis was conducted by the leave-one-out method and checking the consistency of the overall effect estimate. For IL-6, we found that the I^2^ value decreased to 0% after excluding the studies conducted by Shao et al. (2005) and Wu et al. (2013b). For TNF-α, we found that the I^2^ value decreased to 54% after excluding the studies conducted by Tang et al. (2013) and Wang et al. (2007). For IL-10, we found that the I^2^ value decreased to 84% after removing the study by Shao et al. (2005), Wang et al. (2007) and Wu et al. (2013b). We believe that the high heterogeneity may arise from factors such as sample size, different measuring instruments, and design methods”.

In the abstract, “(MD = -88.5, 95% CI [-123.97,-53.04], p < 0.00001)” of IL-6 were changed to “MD = -53.00, 95% CI [-95.56, -10.05], p = 0.02.” “95% confidence interval (CI) [0.35-0.66]” of all-cause mortality was changed to “95% confidence interval (CI) [0.35, 0.66]”. “mean difference (MD) = -2.40, 95% CI [-4.37, -0.44], p = 0.02, I^2^ = 66%” of APACHE II score was changed to “mean difference (MD) = -3.18, 95% CI [-4.01, -2.35], p < 0.00001, I^2^= 33%”. “MD = -56.22, 95% CI [-72.11, -40.33], p < 0.00001” of TNF-α was changed to “MD = -53.05, 95%CI [-68.36, -37.73], p < 0.00001”. The mistake was correlated with the mistake of **Figure 3A**; it has been changed in the corresponding place in the text. A correction has been made to the **Abstract**:

“Results: Ulinastatin significantly decreased the all-cause mortality {odds ratio (OR) = 0.48, 95% confidence interval (CI) [0.35, 0.66], p < 0.00001, I^2^ = 13%}, Acute Physiology, Age, Chronic Health Evaluation II (APACHE II) score {mean difference (MD) = -3.18, 95%CI [-4.01, -2.35], p < 0.00001, I^2^ = 33%, and reduced the incidence of multiple organ dysfunction syndrome (MODS) (OR = 0.3, 95% CI [0.18, 0.49], p < 0.00001, I^2^ = 0%). Ulinastatin also decreased the serum levels of IL-6 (MD = -53.00, 95% CI [-95.56,-10.05], p = 0.02), TNF-α MD = -53.05, 95%CI [-68.36, -37.73], p < 0.00001, and increased the serum levels of IL-10 (MD = 37.73, 95% CI [16.92, 58.54], p = 0.0004). Ulinastatin administration did not lead to any difference in the occurrence of adverse events.”

In the subsection *Outcomes and Data Extraction*, the formula “X = |X_2_ - X_1_|” was changed to “X = X_2_ - X_1_.” The reason for this correction is that the observation index in this study was the effect of ulinastatin for sepsis patients. “X_2_ represents the endpoint SD. R = 0.5” was changed to “S_2_ represents the endpoint SD. R = 0.5.” The mistakes were made due to a typographical error. A correction has been made to the **Materials and Methods** section, subsection **Outcomes and Data Extraction**, paragraph 1:

“The primary outcome was all-cause mortality. The secondary outcomes were changes in the serum levels of IL-6, IL-10, and TNF-α, the incidence rate of MODS, and changes in Acute Physiology, Age, Chronic Health Evaluation II (APACHE II) scores. We also collected the following information: study design, year of study, country, study period, the number of patients included, intervention methods, and adverse events. The main characteristics of the included studies are shown in **Table 2**. For the continuous variables, we acquired data according to the following method. For calculating the mean in this meta-analysis, we employed the formula *X = X*_2_ - *X*_1_, where *X* represents the mean applied in this meta-analysis, *X*_1_ represents the baseline mean, and *X*_2_ represents the endpoint mean. For calculating the standard deviation (SD) in this meta-analysis, we chose to employ the formula *S^2^ =* S_1_^2^ + S_2_^2^ – 2 × R × S_1_ × S_2_, where *S* represents the standard deviation applied in this meta-analysis, S_1_ represents the baseline SD, and *S*_2_ represents the endpoint SD. R = 0.5 in the meta-analysis, which was described in the Cochrane Handbook. All data were independently extracted by two authors (HW and LY). HW entered data into the computer and LY checked them.”

In the subsection “All-Cause Mortality,” p = 0.37 was changed to p = 0.32. The mistake was made due to a typographical error. A correction has been made to the **Results** section, subsection **Primary Outcomes, All-Cause Mortality**, paragraph 1:

“We extracted the data from 12 studies (Fang and Chen, 2005; Shao et al., 2005; Ni et al., 2008; Sung et al., 2009; Tang, 2013; Wu et al., 2013b; Karnad et al., 2014; Chen et al., 2015; Choudhuri et al., 2015; Wu et al., 2016; Fang and Zhao, 2017; Pavan Kumar Rao et al., 2017) and 1110 participants were classified into two groups to assess all-cause mortality. All-cause mortality was significantly lower in the UTI group than in the control group (OR = 0.48, 95% CI [0.35, 0.66], p < 0.00001), and heterogeneity was low (x^2^ = 12.57, p = 0.32, I^2^ = 13%). The results are shown in **Figure 2**.”

In the subsection *Levels of IL-6*, the 402 participants in two groups was changed to 558 participants in two groups. The mistake was made due to a miscalculation. “MD = -88.50, 95% CI [-123.97, -53.04], p < 0.00001” was changed to “MD = -53.00, 95% CI [-95.56,-10.05], p = 0.02”; “x^2^ = 249.27, p < 0.00001, I^2^ = 96%” was changed to “x^2^ = 410.24, p < 0.00001, I^2^ = 98%.” The error was made due to the mistake in **Figure 3A**. A correction has been made to the **Results** section, subsection **Levels of IL-6**, paragraph 1:

“We obtained the related data from eight studies (Fang and Chen, 2005; Shao et al., 2005; Jiang et al., 2006; Wang et al., 2007; Tang, 2013; Wu et al., 2013b; Chen et al., 2015; Fang and Zhao, 2017) (558 participants in two groups) to analyze the serum levels of IL-6. The serum level of IL-6 at the time of hospital admission was not different between the UTI and control groups. After treatment, IL-6 was significantly less in the UTI group than in the control group (MD = -53.00, 95% CI [-95.56,-10.05], p = 0.02), and a obvious heterogeneity in the results was observed (x^2^ = 410.24, p < 0.00001, I^2^ = 98%). The results are shown in **Figure 3A**.”

In the subsection of *Levels of TNF-a*, “MD = −56.22, 95% CI [−72.11, −40.33], p < 0.00001” was changed to “MD = -53.05, 95%CI [-68.36,-37.73], p < 0.00001”; “x^2^ = 47.26, p < 0.00001, I^2^ = 81%” was changed to “x^2^ = 43.58, p < 0.00001, I^2^ = 79%.” A correction has been made to the **Results** section, subsection **Levels of TNF-a**, paragraph 1:

“We collected the related data from ten studies (Fang and Chen, 2005; Shao et al., 2005; Jiang et al., 2006; Wang et al., 2007; Ni et al., 2008; Tang, 2013; Chen et al., 2015; Dai and Wang, 2016; Wu et al., 2016; Fang and Zhao, 2017) (686 participants in two groups) to analyze the serum levels of TNF-α. The level of TNF-α at the time of hospital admission was not different between the UTI and control groups. After treatment, TNF-α was significantly less in the UTI group than in the control group (MD = -53.05, 95%CI [-68.36,-37.73], p < 0.00001), and an obvious heterogeneity was observed in the results (x^2^ = 43.58, p < 0.00001, I^2^ = 79%). The results are shown in **Figure 3B**.”

In the subsection *The Apache Ii Score*, the title should be *The Apache Ⅱ score*. Also in this section, the (298 participants in two groups) was changed to (244 participants in two groups). “MD = −2.40, 95% CI [−4.37, −0.44], p = 0.02” was changed to “MD = -3.18, 95%CI [-4.01,-2.35], p < 0.00001”; “x^2^ = 8.86, p = 0.03, I^2^ = 66%” was changed to “x^2^ = 4.51, p = 0.21,I^2^ = 33%.” The mistake was made by including wrong citation. A correction has been made to the section **Results**, subsection **The Apache Ⅱ Score**, **Title**: **The Apache Ⅱ Score**, paragraph 1:

“The APACHE II scores at the time of hospital admission were not different between the UTI and control groups. We extracted the data from four studies (Ni et al., 2008; Fang et al., 2005; Dai and Wang, 2016; Wu et al., 2016) (244 participants in two groups) to assess this change. After treatment, the APACHE II scores were significantly less in the UTI group than in the control group (MD = -3.18, 95%CI [-4.01,-2.35], p < 0.00001), and heterogeneity was low (x^2^ = 4.51, p = 0.21,I^2^ = 33%). The results are shown in **Figure 4**.”

In the section of **Publication Bias and Sensitivity Analysis**, “74%” was changed to “0%.” “81%” was changed to “54%.” The mistake was made by included wrong study. Also, we deleted the sensitivity for APACHE II score and added IL-10. Because we found that the heterogeneity of APACHE II was 33% and the heterogeneity of IL-10 was 98%. A correction has been made to **Results** section, subsection **Publication Bias and Sensitivity Analysis**, paragraph 2:

“Sensitivity analysis was conducted by the leave-one-out method and checking the consistency of the overall effect estimate. For IL-6, we found that the I^2^ value decreased to 0% after excluding the studies conducted by Shao et al. (2005) and Wu et al. (2013b). For TNF-α, we found that the I^2^ value decreased to 54% after excluding the studies conducted by Tang et al. (2013) and Wang et al. (2007). For IL-10, we found that the I^2^ value decreased to 84% after removing the study by Shao et al. (2005), Wang et al. (2007) and Wu et al. (2013b). We believe that the high heterogeneity may arise from factors such as sample size, different measuring instruments, and design methods.”

The authors apologize for these errors and state that they do not change the scientific conclusions of the article in any way. The original article has been updated.

